# Artificial Intelligence as a Tool for the Diagnosis and Treatment of Neurodegenerative Diseases

**DOI:** 10.3390/brainsci13060938

**Published:** 2023-06-10

**Authors:** George B. Stefano

**Affiliations:** Department of Psychiatry, First Faculty of Medicine, Charles University and General University Hospital in Prague, Ke Karlovu 11, 120 00 Prague, Czech Republic; george.stefano@lf1.cuni.cz

While humans have much in common biologically with other mammalian species, they are largely distinguished by their innate intelligence, specifically, their ability to generate complex and sophisticated tools. This capacity for creativity and ingenuity remains a guiding force in human history and has included such notable events as the invention of the wheel (estimated at 4500–3300 years BCE) and the printing press (mid-1400s CE) and onwards to modern transportation systems, computer technology, robotic automation, and now, artificial intelligence (AI). Collectively, these tools have served to advance the interests and the social evolution of the human species.

The Oxford English Dictionary defines artificial intelligence (AI) as “The capacity of computers or other machines to exhibit or simulate intelligent behaviour (sic); the field of study concerned with this” [1]. This definition highlights an extremely important point. At its best, AI simulates intelligent behavior. In other words, while machine learning engineers can program computers to identify patterns in large datasets and can create programs that permit them to learn from experience, these machines are not intuitive or truly innovative in a human sense. Thus, in a large sense, AI is not substantially different from other disruptive innovations that humans have encountered. At this point in time, no one would argue that transportation systems will replace the ability to walk or that it is somehow better to add columns of numbers manually rather than using a computer to do so. We ultimately need to familiarize ourselves with the basic principles of AI, notably the subset of processes associated with neural networks and deep learning (i.e., the use of multiple layers that permit progressive extraction of higher-level features from the raw input). Readers interested in a basic overview of AI and insights into the future of the field might explore the recent book on this subject [2].

The medical field is poised to make great strides via the use of AI technology. Much has already been accomplished, for example, the ongoing development of decision support algorithms, particularly those involving analyses of visual-pattern-based information, i.e., X-rays, magnetic resonance imaging (MRI), computed tomography (CT), and stained histological samples. At this time, AI is being applied as part of the effort to identify and disentangle more complex patterns that will lead to improvements in diagnosis, treatment decisions, and disease prognosis. In its current and future iterations, AI holds great promise as a tool to improve our understanding of complex progressive neurodegenerative diseases.

Neurodegenerative diseases are a group of disorders in which cells of the central nervous system (CNS) die or deteriorate to the point that they are no longer able to function properly. These disorders may be all or partially genetic and also may be caused by tumors, vascular dysfunction, viral infection, or exposure to toxins.

Of critical note, although there are currently several therapeutic strategies that can be used to manage neurodegenerative disorders, patients typically deteriorate over time and there are no known cures. Thus, one immediate critical use of AI might be to identify critical patterns and pathways involved in the earliest stages of disease pathogenesis. Once these pathways and derangements have been identified, rational strategies might be developed to prevent disease onset and/or limit the extent of otherwise ongoing deterioration. The interested reader is referred to Myszczynska et al. [3] for an informative review of this growing field.

In the paragraphs to follow, we will discuss current efforts to use AI to explore three of the most common neurodegenerative disorders: Alzheimer’s disease (AD), multiple sclerosis (MS), and Parkinson’s disease (PD). 

AD is the most common form of dementia and is a progressive neurodegenerative disease of uncertain etiology that typically begins with mild memory loss and can progress to a near-full loss of cognitive function. A recent report from the United States Center for Disease Control and Prevention [4] cited earlier studies that estimated that 5.8 million Americans 65 years of age or older carried a diagnosis of Alzheimer’s disease in 2020, with projected increases to ~14 million people by 2060. Several medications are currently available for use by patients with AD, including, most recently, lecanemab, a monoclonal antibody that targets specific forms of beta-amyloid proteins [5]. While these medications can be used to manage the symptoms and potentially slow the progression of the disease, none are curative.

The potential of AI as a means to understand this disorder was highlighted in a recent review by Fabrizio et al. [6]. In this publication, the authors discuss the use of AI to evaluate and identify patterns in various large datasets, including co-morbidities, psychological testing, neuroimaging and laboratory findings, and lifestyle issues together with metagenomic and proteomics data collected from individuals who have been diagnosed with AD. Among the primary goals, researchers hope to highlight patterns that can be used to identify individuals presenting with mild cognitive impairment (MCI) who ultimately progress to full-blown AD. Similarly, Frizzell et al. [7] published a systematic review of the use of AI to evaluate brain MR imaging scans of patients with MCI and AD. The authors reviewed the results of 97 published studies and concluded that, among the various AI methods used, the best performance was achieved using deep learning-based convolution neural network algorithms. One example of the results obtained using these methods was reported by McKenzie et al. [8], who used an AI/deep-learning strategy to identify early changes associated with AD. In this study, the authors performed an unbiased examination of histopathological changes that might correlate with cognition in a study of whole slide images of human brain autopsy tissues from a group of elderly donors. Interestingly, they found that the presence or absence of myelin pallor in brain white matter consistently correlated with the presence or absence of cognitive impairment, respectively, in >600 test subjects. Of note, this finding is consistent with reports of others focused on the impact of white matter degeneration in early AD [9,10]. Although these efforts have not yet led to tangible diagnostic or therapeutic insights, they collectively highlight what may be among the earliest detectable changes associated with this neurodegenerative process, and may ultimately lead to targets for specific intervention.

Multiple sclerosis (MS) is a chronic autoimmune disease associated with demyelination and neurodegeneration in the CNS. A recent report by Walton et al. [11] highlighted the increasing prevalence of this disorder, at 2.8 million individuals worldwide in 2020. Clinically, patients with MS experience acute attacks and progressive disability. While numerous disease-modifying drugs and therapies have been developed, none of them can cure this disease. Bonnachi et al. [12] recently reviewed the use of AI to diagnose, predict outcomes, and monitor the treatment of MS based on MRI findings and laboratory results (i.e., blood and CSF levels of active mediators). Among the problems that must be overcome, MS is a heterogeneous disease that can be confused with many other conditions (both inflammatory and non-inflammatory) that present with similar clinical symptoms. Furthermore, a diagnosis of MS relies on evidence of the dissemination of the disease in both time and space. Thus, although AI strategies hold promise for more precise diagnosis and monitoring of diseases, expert human oversight and review of all findings remains essential at this time.

Parkinson’s disease (PD) is a highly prevalent and progressive disorder involving the loss of nigrostriatal dopaminergic neurons [13]. Patients with PD can experience one or more of a constellation of symptoms that include tremors, bradykinesia, hypokinesia, rigidity, and postural instability. Currently, the diagnosis of PD is based on the clinical detection of one or more of these motor symptoms; however, these may only develop after substantial loss of dopaminergic neurons. While signs and symptoms of the disease can be managed with medications, there is no known cure. Recently, Yang et al. [14] published a groundbreaking study in which AI strategies were used that ultimately identified nocturnal breathing disorders as an early objective biomarker of PD. Respiratory symptoms may develop and might be detected many years before clinical motor symptoms emerge [15,16]. Among findings from this AI-based study, the authors found that PD could be predicted and monitored based on individual nocturnal breathing patterns, including those obtained from a formal sleep study and/or from home-based radio frequency detection devices. This will not only lead to early diagnosis, but will also provide researchers with information on new targets and pathways that might be used to improve current therapeutic strategies for PD.

In summary, AI is currently poised to revolutionize the nature of research into complex neurodegenerative diseases and will ultimately have a large impact on clinical practice [17]. By analogy to disruptive technologies of earlier generations, fear and concern will ultimately be minimized once the benefits of this approach become apparent.
